# High Turnover Frequency in the Electrocatalytic Reduction of Nitrous Oxide to Dinitrogen at a Binuclear Copper Complex of 3,5‐Diamino‐1,2,4‐Triazole

**DOI:** 10.1002/anie.202506067

**Published:** 2025-07-02

**Authors:** Zhengwei Ma, Masaru Kato, Jenny Pirillo, Yuh Hijikata, Takeshi Watanabe, Yohei Uemura, Bang Lu, Satoru Takakusagi, Ken'ichi Kimijima, Hideo Notsu, Ichizo Yagi

**Affiliations:** ^1^ Graduate School of Environmental Science Hokkaido University N10W5, Kita‐ku Sapporo 060‐0810 Japan; ^2^ Faculty of Environmental Earth Science Hokkaido University N10W5, Kita‐ku Sapporo 060‐0810 Japan; ^3^ Department of Materials Chemistry, Graduate School of Engineering Nagoya University Furumai‐cho, Chikusa‐ku Nagoya 464‐8603 Aichi Japan; ^4^ Research Center for Net Zero Carbon Society, Institutes of Innovation for Future Society Nagoya University Furo‐cho, Chikusa‐ku Nagoya 464‐8601 Aichi Japan; ^5^ Industrial Application and Partnership Division Japan Synchrotron Radiation Research Institute (JASRI) SPring‐8, 1‐1‐1 Kouto Sayo 679‐5198 Japan; ^6^ FXE instrument European XFEL GmbH Holzkoppel 4 Schenefeld 22869 Germany; ^7^ Institute for Catalysis Hokkaido University N21W10, Kita‐ku Sapporo 001‐0021 Japan; ^8^ Photon Factory, Institute of Materials Structure Science High Energy Accelerator Research Organization 1‐1 Oho Tsukuba 305‐0801 Ibaraki Japan; ^9^ Department of Materials Science and Engineering Institute of Science Tokyo S8‐26, 2‐12‐1 Ookayama Meguro‐ku 152‐8550 Japan

**Keywords:** Denitrification, Electrocatalysis, Metalloenzyme mimic, N_2_O reductase, N_2_O reduction

## Abstract

Nitrous oxide (N_2_O) is a potent greenhouse gas and an ozone‐depleting substance. Electrocatalytic N_2_O reduction (e‐N_2_ORR) is a promising approach to remove N_2_O from the air under ambient conditions. However, developing noble‐metal‐free e‐N_2_ORR electrocatalysts with high Faradaic efficiency (FE) and turnover frequency (TOF) remains a challenge because of the weak binding of N_2_O and the high kinetic barrier for deoxygenation reaction. In this work, inspired by the multinuclear copper active site of nitrous oxide reductases, a binuclear copper complex of 3,5‐diamino‐1,2,4‐triazole supported on carbon black of Ketjenblack (CuHdatrz/KB) was utilized for the e‐N_2_ORR at pH 13 and 298 K. CuHdatrz/KB achieves a high FE of ≈ 100% at −0.3 V versus reversible hydrogen electrode for the e‐N_2_ORR to N_2_ and TOF up to ≈ 700 h^−1^, which is one–two orders of magnitude higher than those of the previously reported molecular‐based catalysts. In situ X‐ray absorption spectroscopy confirmed that Cu(II) ions of CuHdatrz/KB are reduced to Cu(I) keeping the binuclear core, suggesting that the multinuclear copper active site is crucial to efficiently catalyze the e‐N_2_ORR like the nitrous oxide reductase.

## Graphical abstract

Inspired by nitrous oxide reductase, a binuclear copper complex supported by carbon black was utilized for the electrocatalytic N₂O reduction under ambient conditions. The binuclear Cu center enables exceptionally high turnover frequency and nearly 100% Faradaic efficiency for the electrochemical N₂O reduction.

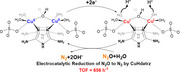

Excess nitrous oxide (N_2_O) from anthropogenic sources disrupts natural denitrification processes (NO_3_^−^→NO_2_^−^→NO→N_2_O→N_2_),^[^ ^1^, ^2^ ^]^ leading to a gradual increase in atmospheric N_2_O levels.^[^ ^3^ ^]^ As of June 2024, the atmospheric N₂O concentration reached 338 ppb, rising by approximately 1 ppb annually.^[^ ^4^ ^]^ N_2_O has a global warming potential ∼300 times higher than that of CO_2_ on a per‐mass basis and is a persistent ozone‐depleting substance.^[^ ^5^, ^6^ ^]^ Without intervention, this trend will worsen.^[^ ^7^ ^]^ Therefore, it is crucial to minimize N_2_O emission and explore effective methods to convert N_2_O into N_2_. N_2_O removal technologies have been developed including thermal decomposition systems, photocatalytic systems, and electrocatalytic systems.^[^ ^8^, ^9^, ^10^, ^11^, ^12^, ^13^ ^]^ Although thermal decomposition efficiently removes N_2_O, the high cost associated with continuous energy input limits its widespread application.^[^ ^14^, ^15^ ^]^ Photocatalytic systems suffer from wide band gaps and rapid charge recombination, significantly limiting their efficiency and scalability, even with advanced modifications such as doping and material coupling.^[^ ^16^, ^17^ ^]^

The electrocatalytic N₂O reduction reaction (e‐N₂ORR) offers a low‐energy, efficient, and scalable alternative to thermal decomposition and photocatalysis for converting N₂O into N₂ or NH_3_ under ambient conditions. Palladium‐based electrocatalysts are particularly effective for the e‐N₂ORR to N₂, with a Tafel slope of 84 ± 7 mV dec^−1^ in 0.1 M NaOH, suggesting potential for further performance enhancement.^[^ ^18^ ^]^ Therefore, strategies such as microstructure modification or alloying have been widely explored.^[^ ^2^, ^11^, ^19^, ^20^ ^]^ Notably, our previous work highlighted that combining palladium with platinum and tin can enhance N₂O adsorption, suppress hydrogen adsorption, and boost e‐N₂ORR activity at palladium‐tin interfaces.^[^ ^2^ ^]^ Nevertheless, palladium's scarcity and cost limit its scalability.

Non‐noble metal‐based electrocatalysts offer a cost‐effective and abundant alternative to noble metal catalysts. However, these catalysts face inherent challenges that limit their performance and adoption. In addition to the inherent difficulty of N_2_O adsorption on metals, attributed to its poor σ‐donating and π‐accepting characteristics, these catalysts must overcome the high dissociation energy of the N─O bond and competitive adsorption from hydrogen.^[^ ^21^, ^22^ ^]^

Biological insights into nitrous oxide reductases provide valuable inspiration for designing advanced electrocatalysts that address the challenges faced by non‐noble metal systems. In nature, many bacteria contain nitrous oxide reductases, which are a multi‐copper metalloenzyme and catalyze the reduction of N_2_O to N_2_ and H_2_O using two electrons and two protons, making this process highly attractive.^[^ ^23^, ^24^ ^]^ There are two copper sites in nitrous oxide reductase (Figure S3): the internal electron transfer site, Cu_A_, and the enzymatic active site, Cu_Z_ (the Cu_4_S cluster).^[^ ^24^ ^]^ The Cu_A_ site receives electrons from cytochrome *c* or cupredoxin and transfers them to the Cu_Z_ site, at which N_2_O is adsorbed and then reduced. In this process, a sulfide bridging ligand of Cu_Z_ delocalizes electrons over the multi‐copper active site, and overcomes the high kinetic barrier for N─O cleavage.^[^ ^25^ ^]^ Structural mimics of the Cu_Z_ site have been reported.^[^ ^26^, ^27^, ^28^ ^]^ For example, a mononuclear copper(II) complex of (2,6‐bis(bis‐2‐*N*‐methylimidazolyl)phosphino)pyridine, Cu‐MeIm_4_P_2_Py, has been reported for the e‐N_2_ORR,^[^ ^29^ ^]^ where mimicking the ligand structure of the reaction center of the nitrous oxide reductase exhibits high e‐N_2_ORR activity with a turnover frequency (TOF) of 54 h^−1^ and a Faradaic efficiency (FE) of 83%.These findings motivated us to study a multinuclear copper complex as a functional mimic of the nitrous oxide reductase.

In this study, we investigated the e‐N_2_ORR activity of a copper complex of 3,5‐diamino‐1,2,4‐triazole (CuHdatrz) in alkaline media. CuHdatrz features the binuclear copper center and is prepared via a one‐step process. Electrochemical measurements of CuHdatrz supported on carbon black of Ketjenblack (CuHdatrz/KB) under N_2_O revealed high e‐N_2_ORR activity and selectivity. In situ X‐ray absorption spectroscopy measurements of CuHdatrz/KB revealed the change of the oxidation state of the metal center and binuclear core structure of CuHdatrz/KB under electrochemical conditions. To elucidate the high e‐N₂ORR activity of CuHdatrz/KB, pH‐dependence of the onset potential for the e‐N_2_ORR and density functional theory (DFT) calculations of its reduced form were also investigated.

CuHdatrz/KB was prepared following a previously reported procedure (Figure 1a).^[^ ^30^ ^]^ KB, CuSO_4_·5H_2_O, and Hdatrz were mixed in Milli‐Q water under ultrasonication, and then stirred overnight at RT. The resulting product was collected under vacuum filtration and then dried under vacuum to obtain CuHdatrz/KB. A ligand of Hdatrz links the two Cu ions. High‐angle annular dark field‐scanning transmission electron microscopy (HAADF‐STEM) images show bright white spots, indicating that Cu atoms are uniformly distributed on the KB surface (Figure 1c). This observation is further supported by energy dispersive X‐ray spectroscopy (EDS) mapping, where the uniform distribution of N and Cu atoms (Figure 1d). The EDS result (Figure S4) confirms an N to Cu atomic ratio of 5:1, demonstrating that CuHdatrz was successfully synthesized and anchored onto the KB surface.

**Figure 1:**
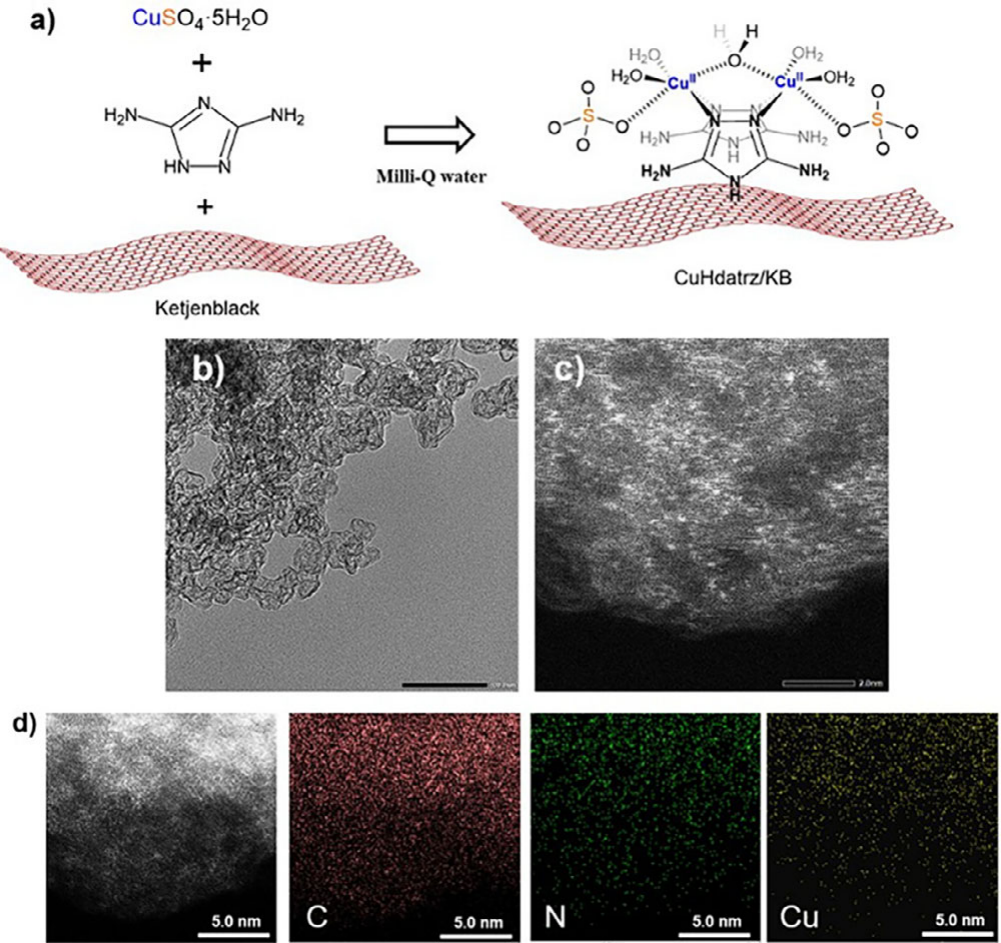
a) The synthesis process of CuHdatrz/KB; b) TEM and c) HAADF‐STEM and EDS mapping images of CuHdatrz/KB.

Cyclic voltammograms (CVs) of CuHdatrz/KB display the potential‐dependence of the competition of the e‐N_2_ORR and hydrogen evolution reaction (HER). CVs in Britton–Robinson (BR) buffered aqueous solutions under Ar (Figure S5) confirmed that CuHdatrz/KB shows the electrocatalytic activity for the HER. In the presence of N_2_O, the onset potential shifted in the positive direction, along with the increase in the current density, resulting from the e‐N_2_ORR by CuHdatrz/KB (Figure S5). As the pH increases, the gap between onset potentials and current densities for the HER and e‐N_2_ORR becomes more pronounced. As shown in Figure 2a, the gap between onset potentials for the e‐N_2_ORR and HER is approximately 0.12 V at pH 2. When the pH increases to 7 and 13, the potential gaps increase to 0.31 and 0.58 V, respectively. The current density differences follow a similar trend, being lowest at pH 2 (1.39 mA cm^−2^), increasing at pH 7 (2.31 mA cm^−2^), and reaching its highest value at pH 13 (3.02 mA cm^−2^) at −0.6 V versus reversible hydrogen electrode (RHE). These results indicate that the e‐N₂ORR becomes more dominant at pH 13 than the HER, justifying subsequent investigations under this condition.

**Figure 2:**
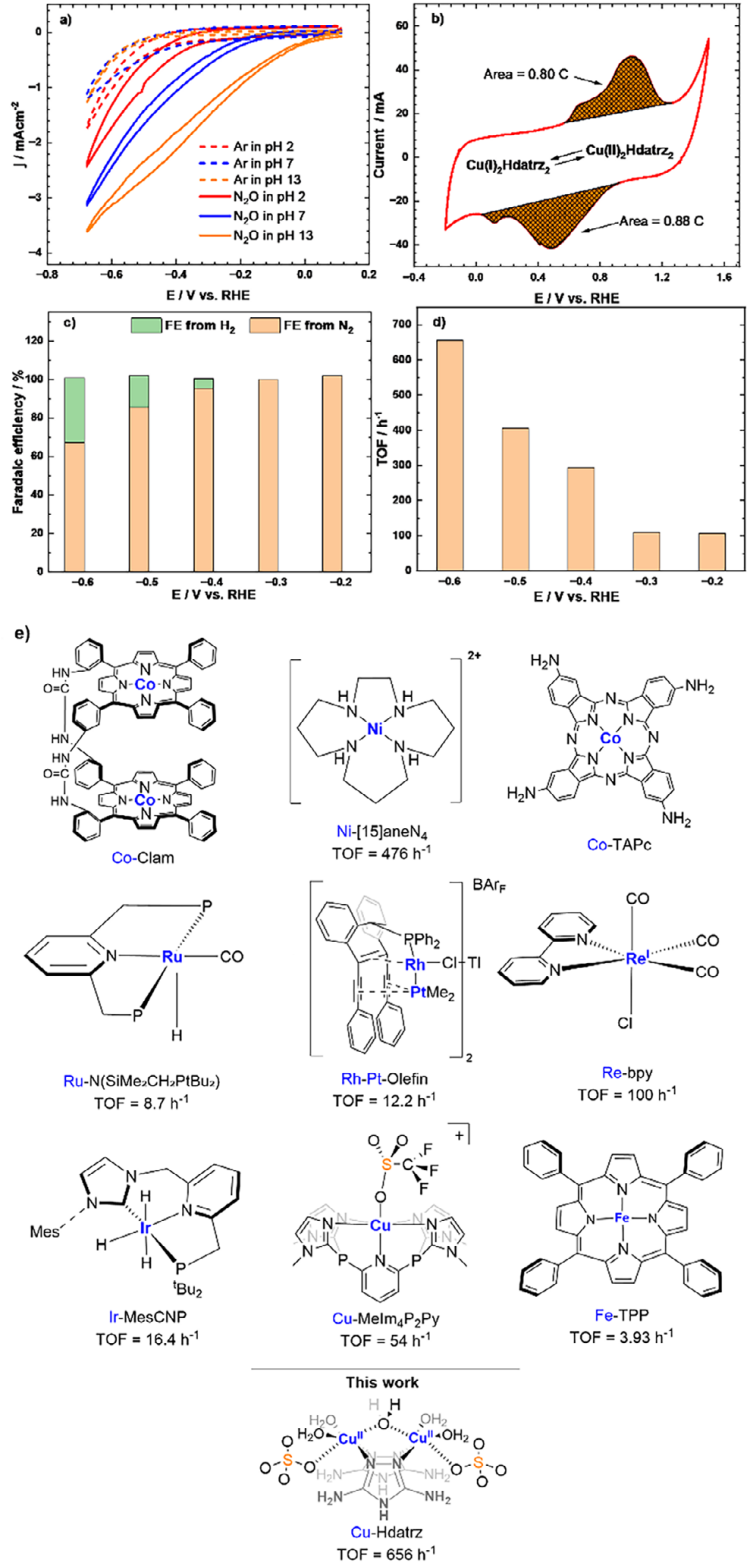
a) CV_S_ of CuHdatrz/KB in BR buffer solutions at pH 2 (red), pH 7 (blue), and pH 13 (orange) under Ar (dot line) and N_2_O (solid line) range from −0.675 to 0.125 V versus RHE, Scan Rate = 10 mV s^−1^, and b) a CV of CuHdatrz/KB under Ar from −0.2 to 1.5 V versus RHE (pH 13, Scan Rate = 10 mV s^−1^). c) FEs and d) TOFs for N_2_ at −0.6, −0.5, −0.4, −0.3, and −0.2 V versus RHE at pH 13. e) Structures and TOFs for the N_2_O reduction of Co‐Clam,^[^ ^38^ ^]^ Ni‐[15]aneN_4_,^[^ ^32^ ^]^ Co‐TAPc,^[^ ^39^ ^]^ Ru‐N(SiMe_2_CH_2_PtBu_2_),^[^ ^33^ ^]^ Rh‐Pt‐Olefin,^[^ ^34^ ^]^ Re‐bpy,^[^ ^35^ ^]^ Ir‐MesCNP,^[^ ^36^ ^]^ Cu‐MeIm_4_P_2_Py,^[^ ^29^ ^]^ and Fe‐TPP.^[^ ^37^ ^]^

CuHdatrz/KB shows its high FE for the e‐N_2_ORR (Figure 2c). FE was determined from chronoamperometry and gas chromatography. For chronoamperometry measurements, a series of potentials (−0.2, −0.3, −0.4, −0.5, and −0.6 V versus RHE) were applied individually to the working electrode (Figure S6), and the generated gases were analyzed using an online gas chromatograph. Throughout the catalytic process for 1 h, the current remained stable at each applied potential, demonstrating high stability of CuHdatrz/KB. N_2_ was the primary product with the FE for N_2_ approaching 100% at potentials of ≥ −0.3 V versus RHE (Figure S7c). At ≤ −0.4 V versus RHE, the e‐N_2_ORR competes with the HER and FE for N_2_ decreases as the potential decreases: 95% FE for N_2_ at −0.4 V; 85% at −0.5 V, and 67% at −0.6 V versus RHE. In contrast, the FE for H_2_ steadily increases: 5.1% at −0.4 V; 17% at −0.5 V; and 33% at −0.6 V versus RHE. Thus, both the HER and e‐N_2_ORR occur simultaneously, and the HER dominates the electrocatalytic process at negative potentials.^[^ ^31^ ^]^ Note that FE for NH_3_ is < 1% (Figure S8) and therefore the main product for the e‐N_2_ORR is N_2_.

CuHdatrz/KB exhibited the highest TOF among reported molecular electrocatalysts. TOFs were determined based on the redox‐active copper ions determined from CVs of CuHdatrz/KB under Ar (∼0.64 µmol cm^−2^, which is 95% of the total loading metal ions) (Figure 2b): 108 h^−1^ at −0.2 V; 110 h^−1^ at −0.3 V; 294 h^−1^ at −0.4 V; 405 h^−1^ at −0.5 V; 656 h^−1^ at −0.6 V versus RHE (Figure 2d). Compared to previously reported TOFs for molecular‐based catalysts, CuHdatrz exhibited the highest TOF (Figure 2e and Table S1).^[^ ^29^, ^32^, ^33^, ^34^, ^35^, ^36^ ^]^ For example, the copper(II) complex of Cu‐MeIm_4_P_2_Py achieved a TOF of 54 h^−1^ (Figure 2e).^[^ ^29^ ^]^ This complex has the single Cu(II) center,^[^ ^28^ ^]^ implying that the binuclear copper center in CuHdatrz is more effective in catalyzing N_2_O reduction. Other metal complexes exhibited TOFs ranging from 3.9 to 476 h^−1^: a nickel complex of 1,4,8,12‐tetra‐azacyclopenta (Ni‐[15]aneN_4_);^[^ ^32^ ^]^ a ruthenium complex of 2,6‐bis(diisopropylphosphino)pyridine (Ru‐N(SiMe_2_CH_2_PtBu_2_));^[^ ^33^ ^]^ a rhodium‐platinum complex of multidentate phosphine olefin (Rh‐Pt‐olefin);^[^ ^34^ ^]^ a rhenium carbonyl complex of 2,2'‐bipyridine (Re‐bpy));^[^ ^35^ ^]^ an iridium complex of 2‐[(Mesitylimidazol‐2‐ylidene)(di‐tert‐butyl)phosphino]pyridine (Ir‐MesCNP);^[^ ^36^ ^]^ an iron complex of tetraphenylporphyrin (Fe‐TPP)^[^ ^37^ ^]^ (Figure 2e). Although cobalt complexes of bis(tetra‐aminophthalocyanine) (Co−Clam)^[^ ^38^ ^]^ and of tetra‐amino‐phthalocyanine (Co‐TAPc)^[^ ^39^ ^]^ were also reported to exhibit high selectivity and activity for the e‐N₂ORR, their TOFs were not provided. The comparison in TOFs between these metal complexes indicates that designing and developing metal complexes with high N₂O reduction activity under ambient conditions remains challenging. Moreover, the TOF of CuHdatrz/KB approaches the range of the natural nitrous oxide reductase enzymes, which achieves TOFs between 63 and 1171 h^−1^.^[^ ^40^, ^41^ ^]^

CuHdatrz/KB exhibited high stability for the e‐N_2_ORR. It maintained a stable current at −0.3 V versus RHE over 24 h, the four 6 h periods. Almost 100% FE remained even after this long‐term experiment and the total turnover number reached to 2656 after 24 h (Figure S9). Inductively coupled plasma optical emission spectrometry measurements of the catalyst film and electrolyte solution confirmed that ∼98% of the initial copper ions remained after the long‐term experiment. Furthermore, almost no change was observed in X‐ray photoelectron spectroscopy (XPS) profiles of CuHdatrz/KB before and after the long‐term experiment (Figure S10). Thus, CuHdatrz/KB is highly durable for the e‐N_2_ORR. Note that the stability of the catalyst film highly depends on the type of binder used: about 30% current loss was observed in CVs under N_2_O after 100 cycles with Nafion whereas an almost negligible decay was observed with polyvinylidene difluoride (Figure S11),^[^ ^42^ ^]^ which was used for the product analysis and long‐term experiments.

An *ex situ* X‐ray absorption spectrum of CuHdatrz/KB in the Cu *K*‐edge X‐ray absorption near edge structure (XANES) region was recorded to understand the initial oxidation state of the Cu center. As shown in Figure 3a, the *ex situ* XANES spectrum of CuHdatrz closely aligns with that of CuO, confirming the presence of the Cu(II) ion in its initial state. This result is consistent with the result of single‐crystal X‐ray analysis.^[^ ^43^ ^]^ The potential‐dependent XANES spectra of CuHdatrz/KB show that Cu(II)Hdatrz is directly converted into a single reduced Cu(I) species under electrochemical conditions without any decomposition: in other words, Cu^II/I^ transformation is reversible (Figure S12).^[^ ^30^ ^]^ These results suggested that the reduction of Cu(II) to Cu(I) species for CuHdatrz is the initial step for the e‐N_2_ORR.

**Figure 3:**
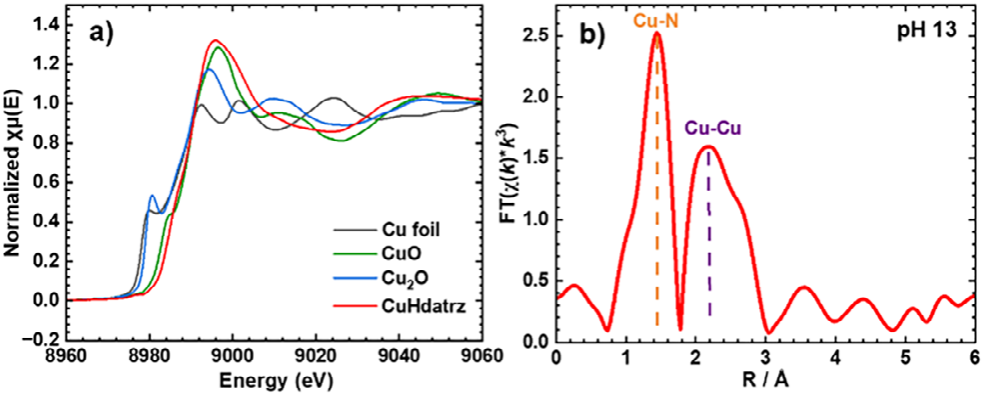
a) Cu *K*‐edge XANES spectra of CuHdatrz and reference samples (Cu foil, CuO, and Cu_2_O), b) FT‐EXAFS oscillation of CuHdatrz/KB at 0.12 V versus RHE at pH 13.

In situ Cu *K*‐edge X‐ray absorption spectroscopy of CuHdatrz/KB was performed at pH 13 and then Fourier transforms of extended X‐ray absorption fine structure (FT‐EXAFS) oscillations were analyzed (Figure 3b). The FT‐EXAFS analysis reveals two distinct peaks assigned to Cu─N and Cu─Cu paths in Cu(I)Hdatrz, highlighting the retention of the binuclear copper structure post‐reduction. The curve‐fitting analysis of the FT‐EXAFS data provides further details on coordination numbers and bond distances: a coordination number of 1.5 and a bond length of 1.85 ± 0.01 Å for the Cu─N path and a coordination number of 0.5 and a bond length of 2.59 ± 0.01 Å for the Cu─Cu path (Table S2), showing a significantly shorter Cu─Cu distance compared to that for CuHdatrz before reduction (3.49 Å).^[^ ^43^ ^]^ In typical Cu(I) complexes, intramolecular Cu─Cu bond distances range from 2.38 to 2.71 Å,^[^ ^44^ ^]^ confirming the presence of the binuclear copper unit of Cu(I)Hdatrz even under reductive conditions. Notably, in nitrous oxide reductases, the interaction with N_2_O induces structural changes in the Cu_Z_ center, adjusting the Cu─Cu distances to enhance N_2_O adsorption and facilitate electron transfer.^[^ ^45^ ^]^ Furthermore, in synthetic Cu_Z_ models, the reduction leads to the Cu_Z_ center adopting a more square geometry with decreasing the distance between 1 and 4 Cu_Z_, responsible for N_2_O adsorption, from 3.0 to 2.8 Å.^[^ ^46^ ^]^ These results collectively highlight the critical role of multinuclear structures, where the dynamic adjustment of metal–metal distances is essential for facilitating catalytic processes. The change in oxidation states of the binuclear Cu center of CuHdatrz, along with the alteration in Cu─Cu distance, resembles the nitrous oxide reductase.

The catalytic behavior of CuHdatrz/KB is strongly influenced by pH. As shown in Figure 4a, in the pH range of 7–10, the slope is 5.9 mV per pH, which is equivalent to −53 mV per pH relative to a pH‐independent potential (Figure S13), closely aligning with the Nernstian prediction of −59 mV per pH.^[^ ^30^ ^]^ This suggests that the rate‐determining step involves the transfer of the same number of electron(s) and proton(s): two electrons and two protons or one electron and one proton. Since the binuclear Cu(II) active site is reduced to Cu(I) during the catalytic process, the rate‐determining step is more likely to involve the transfer of two electrons and two protons.^[^ ^30^, ^47^ ^]^ In strong alkaline media at pH 10–14, the onset potential no longer exhibits a linear dependence on pH (Figure 4a and Figure S13). This change reflects a change in the reaction mechanism for the e‐N₂ORR catalyzed by CuHdatrz, which can be induced by alkaline environments at pH 10–14. It is most likely that deprotonation from ligands contributes to this change.

**Figure 4:**
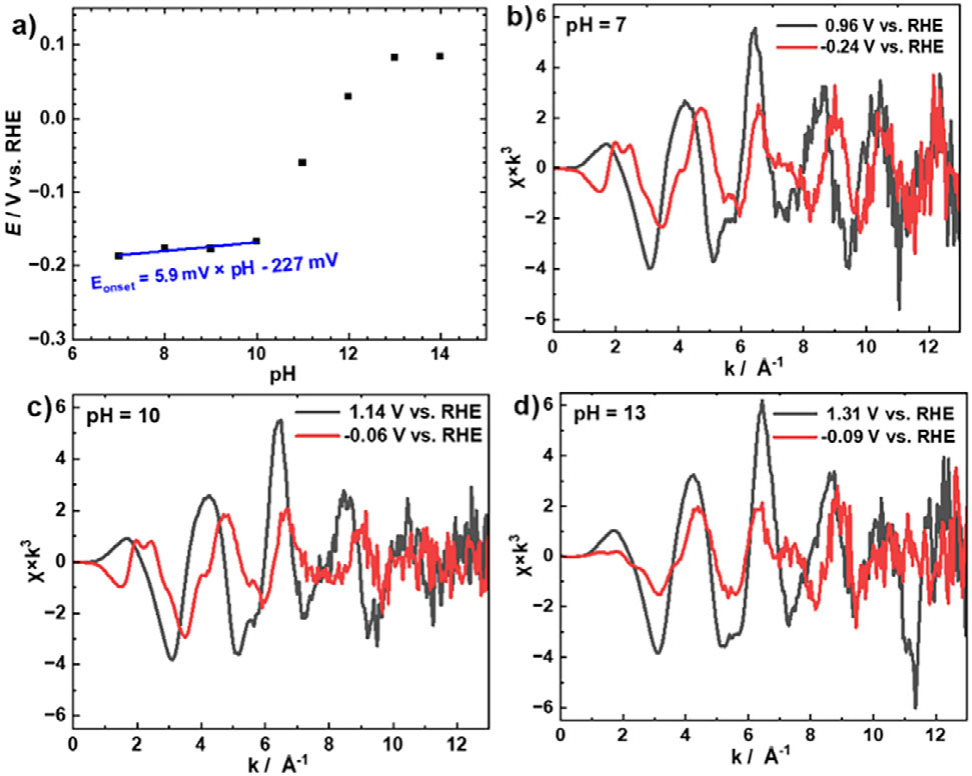
a) Plots of onset potential for the e‐N_2_ORR versus pH. The plots gave a linear relationship with *E*_onset versus RHE_ = 5.9 mV × pH–227 mV in pH 7–10. EXAFS oscillations of CuHdatrz under nitrogen at b) pH 7, c) pH10, and d) pH 13.

EXAFS oscillation profiles of CuHdatrz at different pH provide insights into changes of the local atomic environment in CuHdatrz.^[^ ^48^ ^]^ The EXAFS oscillation profiles at around 2 Å^−1^ show nearly identical oscillations at pH 7 and pH 10, whereas those at pH 13 differ significantly (Figure 4b–d). Differences in the FT‐EXAFS spectra (Figure S14) and curve‐fitting analysis (Table S2) at various applied potentials further illustrate these structural changes. Possibly, the ligand deprotonation occurs at pH 13, altering the electronic structure of CuHdatrz. In DFT calculations, we obtained optimized structures of deprotonated forms with Cu(I) ions and hydroxide ligands (Figure S15), further supporting that the deprotonation from H₂O ligands is more favorable than that of Hdatrz ligands. These results suggest that designing ligands for the multinuclear copper active sites is critical to facilitate the efficient e‐N_2_ORR catalysis and deprotonation of ligands plays a critical role in modulating the activity of CuHdatrz for the e‐N₂ORR.

In conclusion, we demonstrated the e‐N_2_ORR activity of the binuclear copper CuHdatrz/KB under ambient conditions. CuHdatrz/KB achieved the 100% FE with the TOF of 110 h^−1^ at −0.3 V versus RHE and pH 13. Even at −0.5 V versus RHE, showing 85% FE with a TOF of 405 h^−1^. These high FEs and TOFs surpass previously reported molecular electrocatalysts for the e‐N_2_ORR. In situ X‐ray absorption spectroscopy of CuHdatrz/KB revealed that Cu(II) is reduced to Cu(I) and the binuclear copper core remains after the reduction. The high e‐N_2_ORR activity of CuHdatrz/KB especially under strong alkaline (pH 10–14) conditions can be induced by structural changes of CuHdatrz involving the deprotonation of H_2_O molecules, supported by DFT calculations. Our results demonstrate that the multinuclear Cu active site in CuHdatrz/KB is critical for efficient e‐N_2_ORR and illustrates the feasibility of mimicking nitrous oxide reductases with Cu‐based molecular catalysts. Notably, TOFs of natural nitrous oxide reductases (∼29400 h⁻¹ with artificial electron mediators)^[^ ^49^ ^]^ remain far superior to CuHdatrz/KB, highlighting the need for further efforts to develop functionally mimicking Cu‐based molecular catalysts with high e‐N_2_ORR activity.

## Supporting Information

The authors have cited additional references within the Supporting Information.

## Conflict of Interests

The authors declare no conflict of interest.

## Supporting information



Supporting Information

## Data Availability

The data that support the findings of this study are available in the supplementary material of this article.
