# Supplementary material for: High Turnover Frequency in the Electrocatalytic Reduction of Nitrous Oxide to Dinitrogen at a Binuclear Copper Complex of 3,5‐Diamino‐1,2,4‐Triazole
Source: Angew Chem Int Ed Engl. 2025 Jul 2;64(34):e202506067. doi: 10.1002/anie.202506067 (PMC12363637; doi:10.1002/anie.202506067)
Supplement: Supplementary file 1 — Supporting Information [file ANIE-64-e202506067-s001.pdf]

Supporting Information

# High Turnover Frequency in the Electrocatalytic Reduction of Nitrous Oxide to Dinitrogen at a Binuclear Copper Complex of 3,5-Diamino-1,2,4-Triazole

Zhengwei Ma,[a] Masaru Kato,*[a,b] Jenny Pirillo,[c] Yuh Hijikata,[d] Takeshi Watanabe,[e] Yohei Uemura,[f] Bang Lu,[g] Satoru Takakusagi,[g] Ken'ichi Kimijima,[h] Hideo Notsu,[i] Ichizo Yagi*[a,b]

[a] Z. W. Ma, Prof. M. Kato, Prof. I. Yagi
Graduate School of Environmental Science, Hokkaido University, N10W5, Kita-ku, Sapporo 060-0810, Japan

[b] Prof. M. Kato, Prof. I. Yagi
Faculty of Environmental Earth Science, Hokkaido University, N10W5, Kita-ku, Sapporo 060-0810, Japan
E-mail: masaru.kato@ees.hokudai.ac.jp; iyagi@ees.hokudai.ac.jp

[c] Dr. J. Pirillo
Department of Materials Chemistry, Graduate School of Engineering, Nagoya University, Furumai-cho, Chikusa-ku, Aichi, 464-8603, Japan

[d] Prof. Y. Hijikata
Research Center for Net Zero Carbon Society, Institutes of Innovation for Future Society, Nagoya University, Furo-cho, Chikusa-ku, Nagoya, Aichi 464-8601, Japan

[e] Dr. T. Watanabe
Industrial Application and Partnership Division, Japan Synchrotron Radiation Research Institute (JASRI), SPring-8, 1-1-1 Kouto, Sayo 679-5198, Japan

[f] Dr. Yohei Uemura
FXE instrument, European XFEL GmbH, Holzkoppel 4, 22869 Schenefeld, Germany

[g] Dr. Bang Lu, Prof. Satoru Takakusagi
Institute for Catalysis, Hokkaido University N21W10, Kita-ku, Sapporo 001-0021, Japan

[h] Dr. Ken'ichi Kimijima
Photon Factory, Institute of Materials Structure Science, High Energy Accelerator Research Organization, 1-1 Oho, Tsukuba, Ibaraki, 305-0801, Japan

[i] Dr. Hideo Notsu
Department of Materials Science and Engineering, Institute of Science Tokyo, S8-26 , 2-12-1 Ookayama Meguro-ku 152-8550, Japan

## Experimental Section

### Material

$CuSO_4{\cdot}5H_2O$ (99.5%), iso-propanol, and 5% Nafion 117 dispersion were purchased from Wako Pure Chemical Industries Ltd. Polyvinylidene difluoride (PVDF) and 3,5-diamino-1,2,4-triazole was commercially available from Sigma-Aldrich Co. LLC. Ketjen black ECP 600 as a carbon support was purchased from Lion Specialty Chemicals CO. LTD. *N*,*N*-Dimethylformamide (DMF) was purchased from Junsei Chemical Co., Ltd. Ultrapure argon (99.9995%, Hokkaido Air Water Inc.) was used to remove dissolved oxygen from the electrolyte solutions. and nitrous oxide (99.99%, Hokkaido Air Water Inc.) was used as the reactant. $NaClO_4$ (>97%, Kanto Chemical Co Inc), $H_3PO_4$ (85%, Guaranteed Reagent, Sigma-Aldrich Co. LLC), $H_3BO_3$ (Guaranteed Reagent, Wako Pure Chemical Industries Ltd.), acetic acid (Guaranteed Reagent, Wako Pure Chemical Industries Ltd.) and NaOH (Guaranteed Reagent, Wako Pure Chemical Industries Ltd.) were used to prepare electrolyte solutions. For $NH_3$ detection, phenol (>99%, Wako Pure Chemical Industries Ltd.), trisodium citrate (>99%, Wako Pure Chemical Industries Ltd.), and sodium hypochlorite (Practical Grade, Wako Pure Chemical Industries Ltd.) were used to quantify ammonia. Milli-Q ultrapure water from a filtration system (MILLIPORE, USA) and $CH_3CH_2OH$ from Japan Alcohol Trading Co. Ltd. were used for solution preparation.

### Catalyst Preparation

CuHdatrz/KB was prepared according to a method described in earlier studies.[1] Ketjen Black ECP 600, $CuSO_4 \cdot 5H_2O$, and Hdatrz were mixed in Milli-Q water, sonicated, and stirred overnight at room temperature. The resulting product was filtered and vacuum-dried to obtain CuHdatrz/KB.

## Electrolyte Preparation

Britton-Robinson (BR) buffer solutions containing 0.1 M $NaClO_4$ were used as electrolyte solutions at pH 2–13. To prepare electrolyte solutions at pH 2-13, $H_3PO_4$ (4.61 g, 0.04 mol), $CH_3COOH$ (2.40 g, 0.04 mol), $H_3BO_3$ (2.47 g, 0.04 mol) and $NaClO_4$ (12.2 g, 0.1 mol) were dissolved in Milli-Q water. The solution mixture was mixed thoroughly and diluted to 1 L to obtain 0.04 M BR buffer stock solutions. Next, NaOH (8 g, 0.2 mol) and $NaClO_4$ (12.2 g, 0.1 mol) were dissolved in Milli-Q water and then diluted to 1 L as alkaline solution. The pH of the BR buffered aqueous solutions (pH 2-13) were adjusted using the alkaline solution, monitoring the pH using a pH meter for accuracy. As an electrolyte solution at pH14, NaOH (44 g, 1.1 mol) and $NaClO_4$ (12.2 g, 0.1 mol) were dissolved in Milli-Q water and diluted to 1 L adjusting to pH 14.

## Electrochemical Measurements

All electrochemical data were recorded using a potentiostat (HZ7000, Hokuto Denko Corp) with a high-current booster (HZAP-3003A, Hokuto Denko Corp.) in a standard three-electrode electrochemical cell (VB12A, EC Frontier Co., Ltd). An Ag|AgCl electrode (International Chemistry Co., Ltd.) in a saturated KCl solution served as the reference electrode, while a carbon rod (Φ 3 × 100 mm, BAS Inc.) was used as the counter electrode. All potentials were referenced to the reversible hydrogen electrode (RHE) using the Eq (1):

$$E_{RHE} = E_{Ag|AgCl} + 0.198 + pH \times 0.059 \qquad (1)$$

The electrochemical cell was thoroughly cleaned by rinsing with a mixture of $HNO_3$ and $H_2SO_4$ in a 1:1 volume ratio, followed by boiling water at least three times before use. Glassy carbon (GC) plates (25 × 25 × 2 mm³, ALS Co., Ltd.) were polished with two grades of alumina slurry (0.3 µm and 0.05 µm, Baikalox), rinsed with Milli-Q water under ultrasonication, and dried before applying the catalyst ink. A tape (N-380, Nitto Denko) with 8 mm diameter holes was used to mask the surface of the GC, defining a geometrical surface area of 0.50 cm² for the catalyst deposition.

The catalyst ink was prepared by mixing 20 mg of CuHdatrz/KB with 1.6 mL of Milli-Q water, 0.4 mL of $CH_3CH_2OH$, and 30 µL of 5% Nafion dispersion, which was used as a binder. The mixture was sonicated for 30 minutes. Then, 40 µL of the catalyst ink was drop-cast onto the GC, which was then dried at 40 ºC for 30 minutes and subsequently heated to 160 ºC for 5 minutes to ensure strong adhesion. Afterward, the tape was removed, and the GC was placed at the bottom of the cell.

Cyclic voltammograms (CVs) were recorded in BR buffer solutions with pH values ranging from 2 to 13. CVs were recorded in the NaOH aqueous solution at pH 14. Prior to CV measurements, the electrolyte solution was purged with Ar for at least 30 minutes. For electrochemical cleaning, 10 potential cycles were conducted at a scan rate of 100 mV $s^{-1}$ over the potential range of -0.676 to +0.113 V vs. RHE (pH 2-14) under Ar. After that, CVs were recorded at a scan rate of 10 mV $s^{-1}$. For electrochemical $N_2O$ reduction, the electrolyte solution in the electrochemical cell was saturated with $N_2O$ gas by bubbling at a flow rate of 9.8 mL $min^{-1}$ for 30 minutes. CVs were then recorded under $N_2O$ at a sweep rate of 10 mV $s^{-1}$.

## Product analysis

Product analysis was conducted in a standard three-electrode electrochemical cell (VB12A, EC Frontier Co., Ltd) (**Figure S1**). The cathodic and anodic compartments were each filled with 28.5 mL of electrolyte, and the two compartments were separated using an anion exchange membrane (FUMASEP®FAA-3-PK-130, FUMATECH BWT GmbH). The counter electrode and reference electrode were the same as those mentioned in the section of Electrochemical measurements. For the working electrode, 2 mL of the ink was uniformly dropped on both sides of carbon sheet (2.5×2.5 $cm^2$) and dried at 65 ºC overnight at room temperature. Note that PVDF as used as a binder for product analysis experiments because the use of PVDF improved the catalyst film stability on the electrode substrate rather than Nafion.[2] For the preparation of the catalyst ink for product analysis, 4 mg of CuHdatrz/KB and 0.5 mg of PVDF were dispersed in 2 mL of NMP, followed by ultrasonication for 30 min.

In the cathodic compartment, an outlet allowed the gases produced by the catalyst, as well as the gases introduced from the inlet above, to be directed to the online micro-gas chromatograph (Micro-GC, 990 Micro GC Agilent) for analysis. The system was operated with Ar as the carrier gas at a pressure of 0.5 MPa. The injector and column temperatures were both maintained at 100 ºC to ensure stable thermal conditions throughout the analysis. A 40 ms injection time was used to introduce the sample into the system. Gas collection began once the current stabilized, with 8-10 sampling cycles performed for each analysis.

A mixed gas of 9.8 $cm^3 \cdot min^{-1}$ Ar and 1 to 3.8 $cm^3 \cdot min^{-1}$ $N_2O$ was used for calibration. In the chromatographic curve, the peak area of $N_2$ displayed a linear relationship with the flow rate.

The numbers of moles ($n$) of $N_2$ and $H_2$ were calculated using the following Eqs. (2) and (3):

$$n\_N_2 = \frac{Q_{flow}\_N_2 \times P \times t}{R \times T} \qquad (2)$$

$$n\_H_2 = \frac{Q_{flow}\_H_2 \times P \times t}{R \times T} \quad (3)$$

where $n\_N_2$ and $n\_H_2$ is the amount of $N_2$ and $H_2$ in moles (mol), respectively, $Q_{flow}\_N_2$ and $Q_{flow}\_H_2$ are the $N_2$ and $H_2$ flow rate ($cm^3$ $min^{-1}$), $P$ is the gas pressure (10.1325 $N \cdot cm^{-2}$), $t$ is the catalytic process time (60 min), $R$ is the universal gas constant (8.314 J $mol^{-1} \cdot K^{-1}$), and $T$ is the temperature in Kelvin (298 K).

To quantify ammonia ($NH_3$), calibration curves were prepared using a colorimetric method involving phenol and sodium nitroprusside. A phenol solution was prepared by diluting 11.1 mL of liquified phenol (≥89%) with 95% v/v $CH_3CH_2OH$ to a final volume of 100 mL. Sodium nitroprusside was prepared as a 0.5% w/v solution. An alkaline citrate solution was made by dissolving 200 g of trisodium citrate and 10 g of sodium hydroxide in deionized water. For the oxidizing solution, 100 mL of the alkaline citrate solution was mixed with 25 mL of a commercial sodium hypochlorite solution (approximately 5%). The assay procedure involved adding 1 mL of the phenol solution, 1 mL of the sodium nitroprusside solution, and 2.5 mL of the oxidizing solution sequentially to a 25 mL sample, with thorough mixing after each addition. The samples were then covered with plastic wrap or paraffin film and allowed to develop color at room temperature (295-300 K) for at least 60 min. The developed color was stable for up to 1440 min. Absorbance was measured at 640 nm using ultraviolet (UV) spectroscopy.

Additional calibration was performed to determine the exact $NH_3$ yield. Absorbance at 640 nm was recorded for $NH_3$ solutions with concentrations of 5 μmol/L, 10 μmol/L, 25 μmol/L, 50 μmol/L, 100 μmol/L, and 250 μmol/L. A clear linear relationship was observed between concentration and absorbance, allowing the calculation of $NH_3$ yield based on this calibration.
The number of moles ($n$) of $NH_3$ was calculated using the following Eq. (4):

$$n\_NH_3 = C \times V \quad (4)$$

where $n\_NH_3$ is the amount of $NH_3$ in moles (mol), $C$ is the concentration ($mol \cdot L^{-1}$), $V$ is the volume of the electrolyte in cell (28.5 mL).

The Faradaic efficiency ($FE$) was calculated using the following Eq. (5):

$$FE = \frac{n \times F \times z}{Q_{\mathrm{charge}}} \times 100\% \quad (5)$$

where $n$ is the number of moles of products, $F$ is Faraday constant (96485 $C \cdot mol^{-1}$), $z$ is the charge associated with the formation of the desired product (2 for $N_2$ or $H_2$ and 8 for $NH_3$), and $Q_{charge}$ is the total charge passed during the experiment (C).

The TOF in the cathodic chamber after 1 hour of electrolysis was determined using Eq. (6):

$$TOF = \frac{n}{n\_CuHdatrz \times t} \quad (6)$$

where $n$ is the amount of production in moles (mol) from analysis, $n\_CuHdatrz$ is number of moles participate in catalytic process, and $t$ is the time (1 h).

The turnover number (TON) in the cathodic chamber after each 360 min to evaluate durability was determined using Eq. (7):

$$TON = \frac{I \times T}{n\_CuHdatrz \times F \times z} \times \frac{FE}{100\ \%} \quad (7)$$

Where $FE$, $n\_CuHdatrz$, $F$ and $z$ are same as mentioned before, and here FE is 100%. $I$ (A) is the average current of each period, and $T$ (21600 s) is the duration of each period.

For the long-term experiment, we performed chronoamperometry measurements at a constant bias potential of –0.3 V vs. RHE. At every 6 h, the gaseous products were quantified using the gas chromatography and the electrolyte solution was replaced by a fresh one.

## Physicochemical measurements

Scanning transmission electron microscopy (STEM) and high-angle annular dirk field (HAADF-STEM) images were recorded using a JEM ARM200F NEOARM atomic resolution analytical electron microscope operated at an accelerating voltage of 200 kV.

X-ray photoelectron spectroscopy (XPS) measurements were conducted with the TX400 X-ray source and RESOLVE 120 spectrometer (PSP Vacuum Technology Ltd, UK) under $2 \times 10^{-7}$ Pa. The Al-Kα (1486.6 eV) line was used as an X-ray source. The X-ray source emission current was set to 10 mA, while its voltage was set to 14 KV. The sample powder was fixed with the double-side carbon tape that attached on a nickel plate. The binding energies (BE) were calibrated by C1s at 284.8 eV.

Inductively coupled plasma optical emission spectrometry (ICP-OES) was conducted with an Agilent 5900 ICP-OES. The catalyst film and electrolyte solution after the long-term experiment for 24 h were used for the sample preparation. The catalyst-coated carbon paper was immersed in DMF for 1 h and subsequently ultrasonicated to disperse the catalyst into the solvent. To recover any remaining catalyst, the carbon paper was rinsed three additional times with fresh DMF. The combined DMF solutions were dried at 65 °C under vacuum, yielding 4.14 mg of recovered material. Next, 1 mg of the recovered catalyst was dispersed in 50 mL of 1 M $HNO_3$, followed by 30 minutes of ultrasonication and continuous stirring for 72 h to ensure complete dissolution. A 1 mL aliquot of the resulting solution was then diluted to 10 mL with Milli-Q water, adjusting the concentration to 0.1 M $HNO_3$ for ICP-OES analysis. To quantify the leached Cu in the electrolyte, 1 mL of electrolyte was collected at the end of each period during the 24 h durability test. Each sample was subsequently diluted tenfold with 0.1 M $HNO_3$ and analyzed by ICP-OES to determine the Cu concentration in solution.

## X-ray absorption spectroscopy

Cu K-edge X-ray absorption spectroscopy (XAS) was performed at the BL14B2 beamline at SPring-8[3]. XAS data were collected using the transmission method for CuHdatrz, a pellet of CuHdatrz diluted in boron nitride (BN), and reference samples (CuO and $Cu_2O$ diluted in BN, as well as Cu foil). For the catalysts, XAS data were obtained using the fluorescence method with a 19-element germanium (Ge) solid-state detector (SSD). The initial oxidation state of Cu in CuHdatrz was determined by comparing the catalyst with the reference samples.

For sample preparation, 40 µL of catalyst ink was drop-cast onto a carbon sheet (PERMA-FOIL PF-20UHP; 0.2 mm thick; TOYO TANSO) with a defined geometrical area of 0.50 $cm^2$ (a circle with an 8 mm diameter). A tape (N-380, Nitto Denko) with a hole of the same size was used to control the deposition area. After removing the tape, the catalyst-coated carbon sheet was heated at 313 K for 30 minutes, followed by heating at 433 K to ensure firm adhesion of the catalyst to the surface.

For XAS measurements using the fluorescence method at the BL12C beamline in PF-KEK, the catalyst-coated sheet was placed in a home-build spectroelectrochemical flow cell and positioned at a 45º angle relative to the X-ray beam and the SSD (**Figure S2**). A GC rod (*R*-3, 3 mm φ, BAS) and an Ag|AgCl electrode (Innovative Instruments, Inc.) with a double junction holder were used as the counter and reference electrodes, respectively. Before in situ XAS measurements, a BR buffer aqueous electrolyte solution (pH 13) was purged with $N_2$ for at least 30 min. XAS spectra of the catalysts were obtained under 1.29 V, 0.67 V, 0.39 V, and 0.09 V vs. RHE. Extended X-ray absorption fine structure (EXAFS) oscillation values, χ(k), were extracted from the XAS data in the k range of 3 to 12 $Å^{-1}$ using REX2000 software package (Rigaku).[4] The pre-edge line and the post-edge background were estimated using a Victoreen function and a smoothing spline curve with the Cook & Sayers criteria respectively.[5] After subtracting the pre- and post-edge line, $k^3\chi(k)$ values were Fourier transformed into R-space. EXAFS fittings were performed using Larch.[6] To extract structural parameters, theoretical standards were computed using FEFF8.2.[7] A goodness of a curve fitting using *R*-factor.[8]

## Computational details

Density functional calculations were performed using Gaussian 16 Rev. C01.[9] All optimizations were performed with the M06 functional.[10] Basis set of cc-pVTZ for C, H, N and O of water, aug-cc-pVTZ for S and O of $SO_4^{2-}$, and SDD for Cu, where the core electrons were replaced with effective core potentials was employed.[11] For the optimization of CuHdatrz, we referred to the single crystal structure of CuHdatrz.[12] Using the optimized structure of CuHdatrz, we optimized two structures at oxidated state of Cu, where we removed two proton atoms from two water molecules. The integral equation formalism polarizable continuum model (IEFPCM) was employed for implicit solvent (water).[13–17]

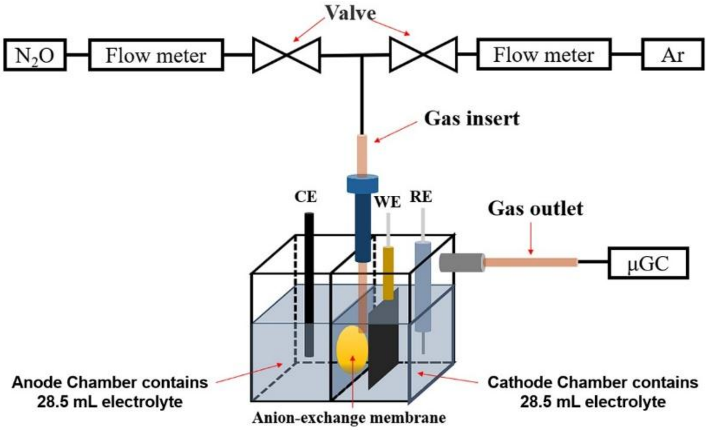


**Figure S1.** A schematic drawing of the electrochemical cell setup for e-$N_2$ORR production detection.

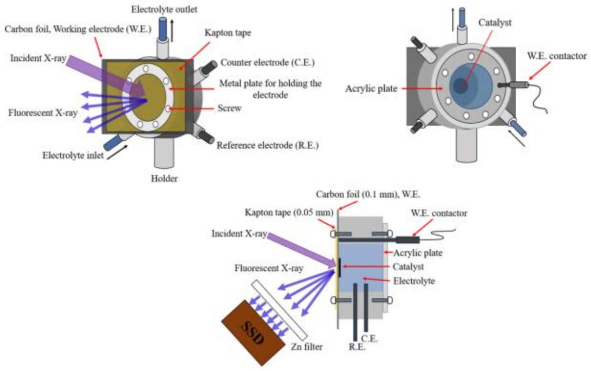


**Figure S2.** Cartoon-like images of our Spectro-electrochemical flow cell for in situ XAS measurements from the front or back side and the top view.[3]

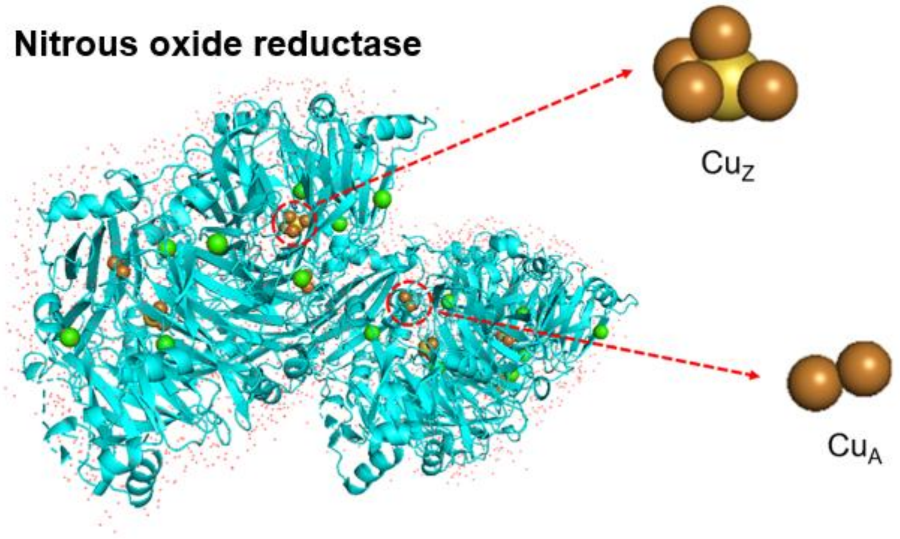


**Figure S3.** Crystal structure of nitrous oxide reductase ($N_2OR$, PDB: 1FWX) highlighting CuA and CuZ centers.

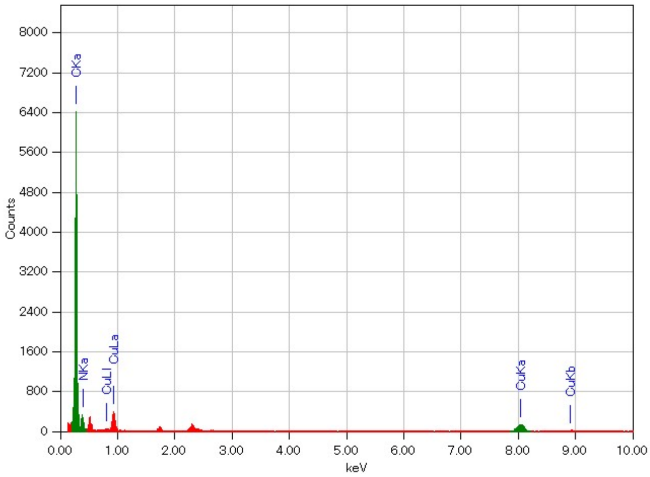


**Figure S4.** EDS spectrum of CuHdatrz/KB, displaying the atomic counts with distinct peaks for Cu, N, O, and C elements.

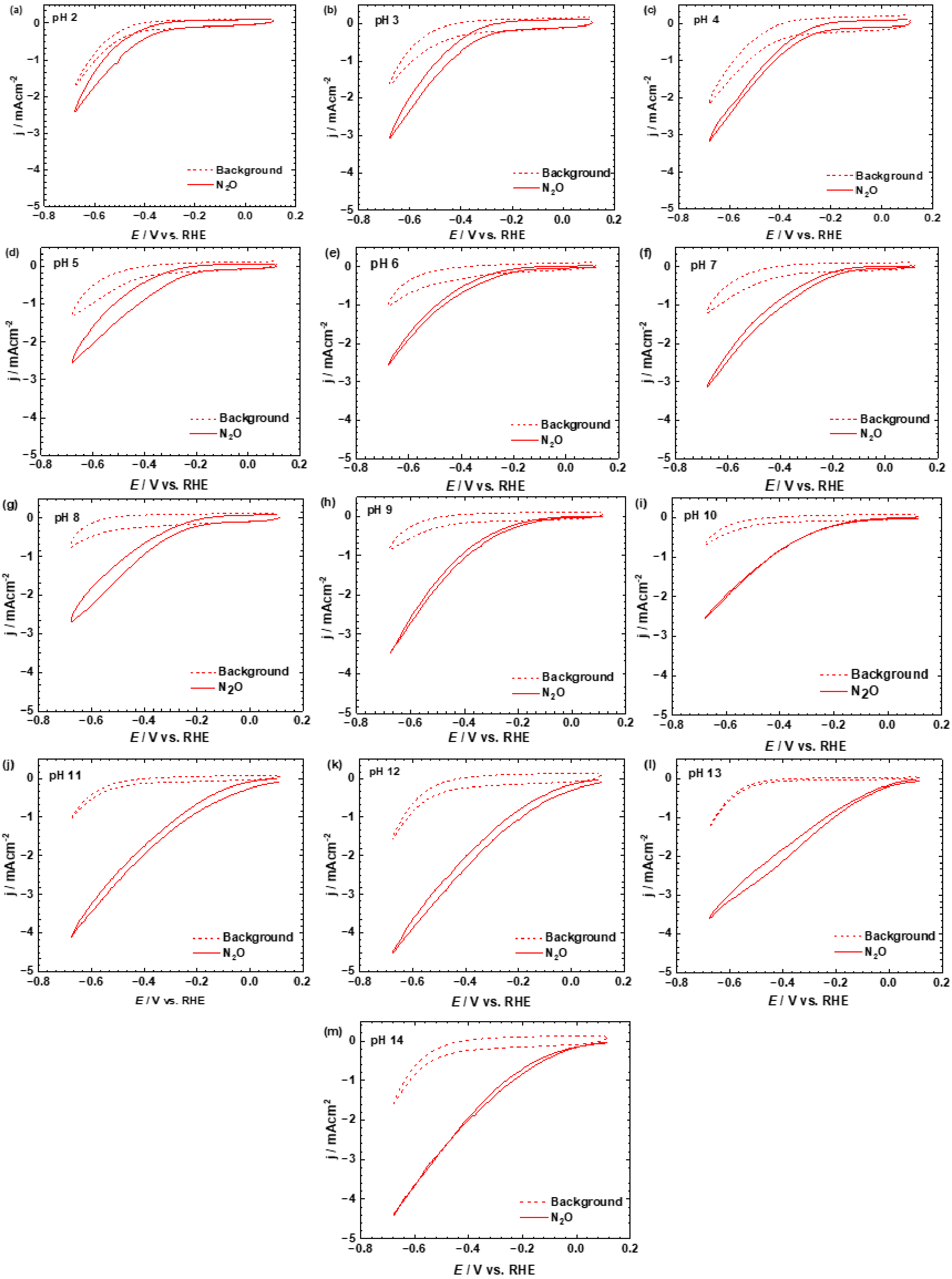


**Figure S5.** CVs of CuHdatrz/KB ranging from 2 to 14 under Ar (dot line) and $N_2O$ (solid line) with the potential range from -0.675 to 0.125 V vs. RHE at a sweep rate of 10 mV $s^{-1}$.

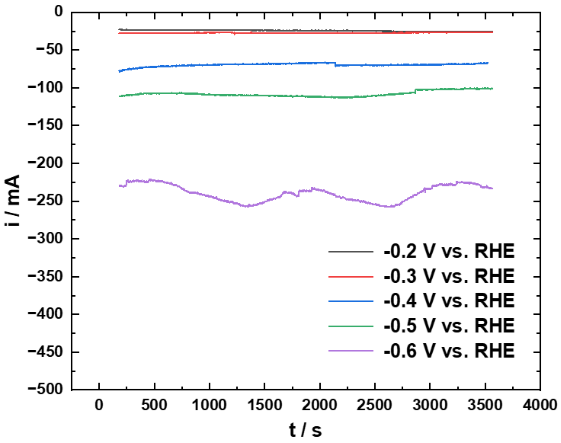


**Figure S6.** Chronoamperometry Curves of CuHdatrz/KB at -0.6 V, -0.5 V, -0.4 V, 0.3 V and -0.2 V vs. RHE in the electrolyte solution at pH 13 under $N_2O$.

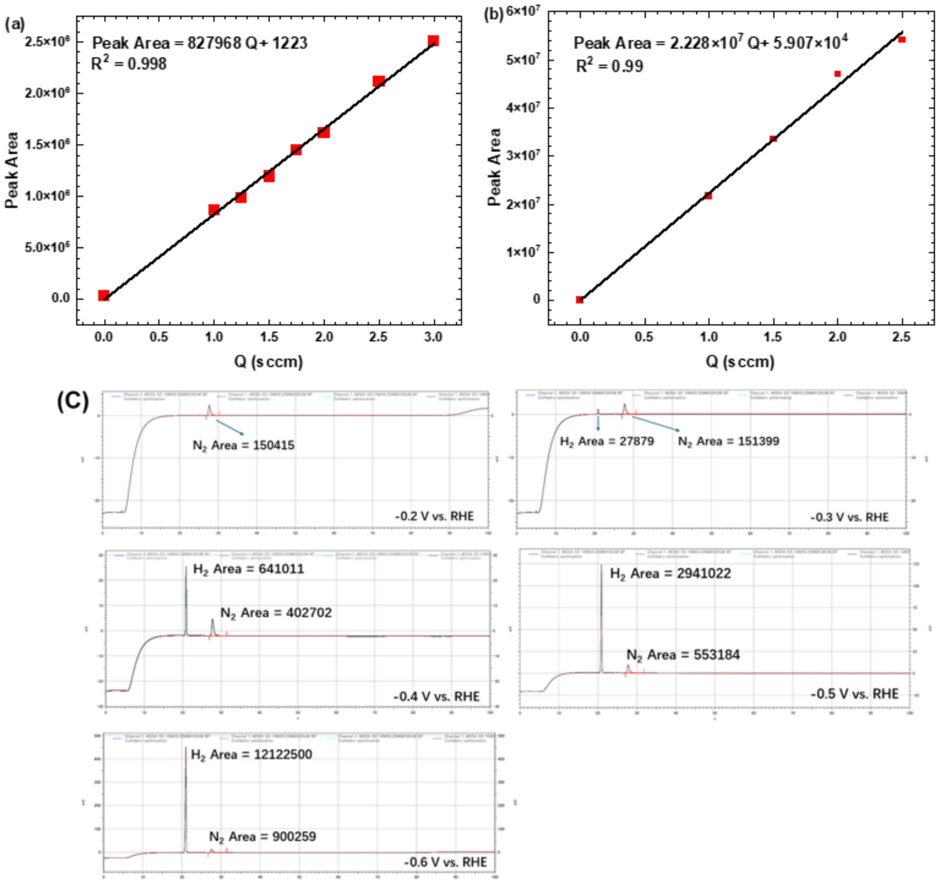


**Figure S7**. Calibration obtained from peak areas at different (a) $N_2$ flow rates and (b) $H_2$ flow rates, (b) Thermal Conductivity Detector peaks of $H_2$ and $N_2$ in GC

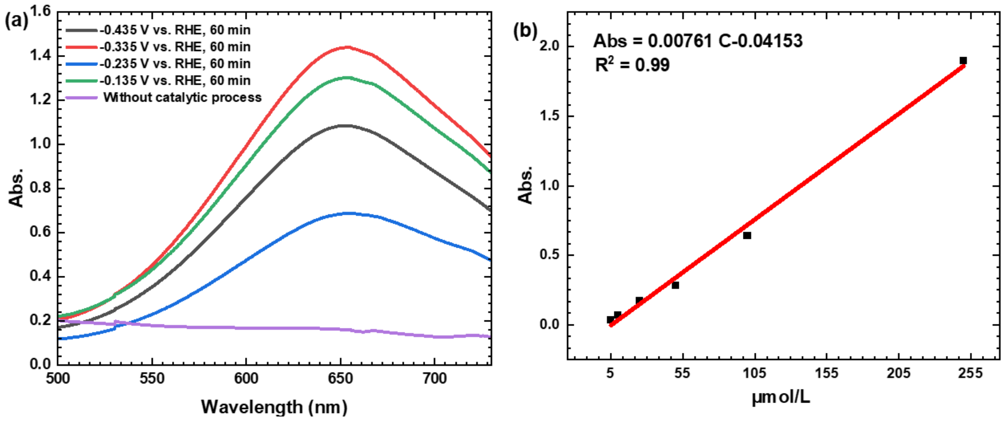


**Figure S8**. (a) UV-Vis absorption spectra of samples after 2 hours of incubation at room temperature. (b) Calibration curve utilized for calculating $NH_3$ concentrations[18].

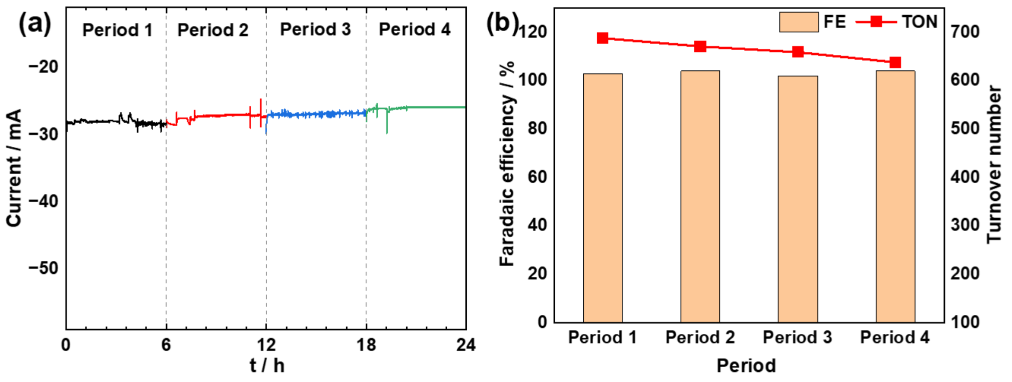


**Figure S9**. (a) Chronoamperometry curve at -0.3 V vs. RHE and pH 13 over 24 h, divided into four equal periods (Average current: 28.8 mA in period 1, 27.3 mA in period 2, 26.9 mA in period 3 and 26.0 mA in period 4). The electrolyte was refreshed at the end of each period to minimize the influence of electrolyte variation. (b) FE and TONs for the e-$N_2$ORR to $N_2$ for each 6 h period during the long-term electrolysis. TONs were determined to be 688 (Period 1), 671 (Period 2), 659 (Period 3), and 638 (Period 4). The total TON reached 2656. ICP-OES results revealed that CuHdatrz/KB retained 98% of its initial copper content after 24 h of electrolysis, with negligible Cu leaching into the electrolyte.

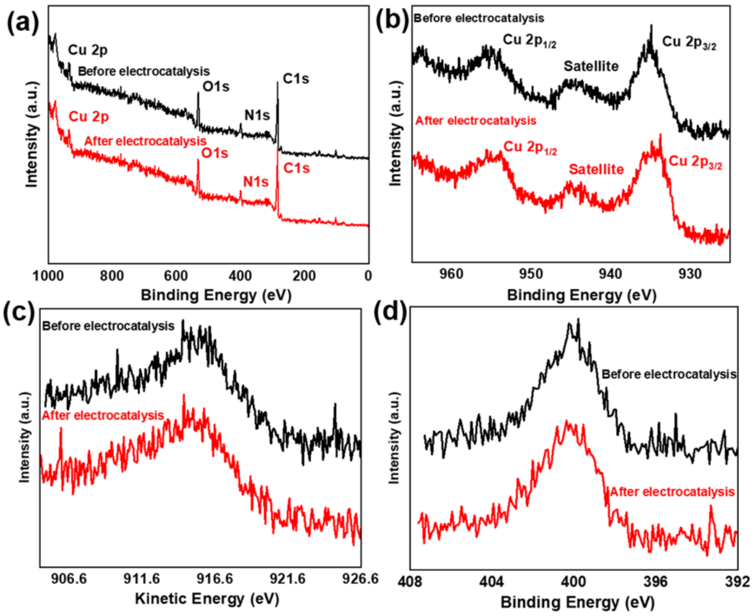


**Figure S10**. XPS data of CuHdatrz/KB before (in black) and after (in red) the long-term electrolysis at -0.3 V vs. RHE and pH 13 for 24 h. (a) Survey XPS profile of CuHdatrz/KB showing the Cu 2p, O 1s, N 1s, and C 1s signals. Magnified XPS profiles of CuHdatrz/KB in the (b) Cu 2p, (c) Cu LMM Auger, and (d) N 1s regions. The Cu oxidation state and nitrogen coordination environment remained essentially unchanged before and after electrolysis, indicating excellent structural stability of the catalyst under reaction conditions.

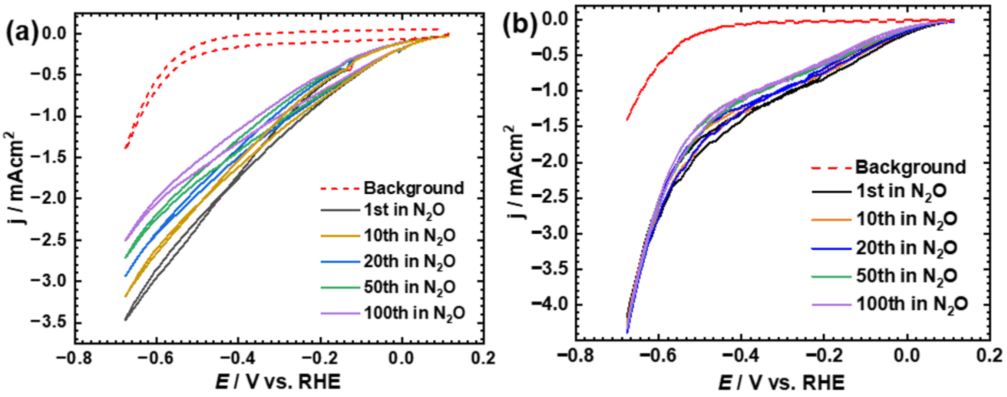


**Figure S11**. Stability evaluation of CuHdatrz/KB for the e-$N_2$ORR. (a) CVs of CuHdatrz/KB using Nafion as binder and (b) using PVDF as binder, recorded in Ar- (the broken lines in red) and $N_2$O-saturated BR buffer aqueous solutions at pH 13 at the 1st (the solid lines in black), 10th (the solid lines in brown), 20th (the solid lines in blue), 50th (the solid lines in green), and 100th (the solid lines in purple) cycles.

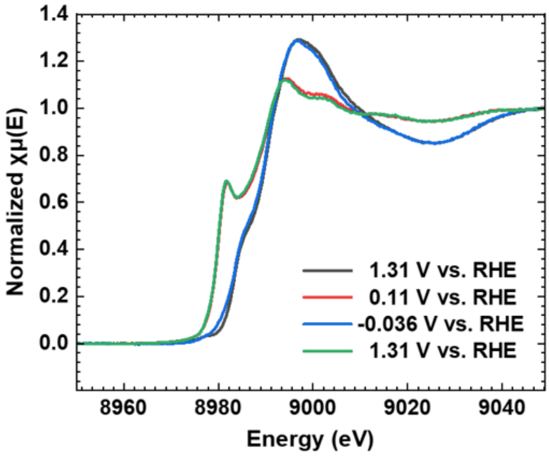


**Figure S12.** *In situ* Cu *K*-edge XANES spectra of CuHdatrz/KB at +1.31 V (in black), 0.11 V (in red), -0.036 V (in blue) and back to +1.31 V (in green) vs. RHE at pH 13.

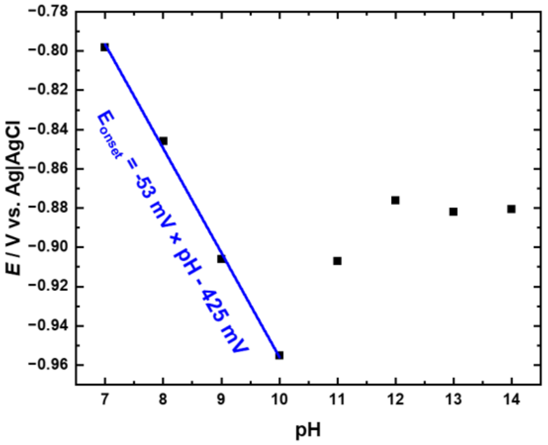


**Figure S13.** Plots of onset potential vs. RHE for e-$N_2$ORR vs. pH. The plots gave a linear relationship with $E_{onset\ vs.\ Ag|AgCl}$ = -53 mV × pH - 425 mV in pH 7-10.

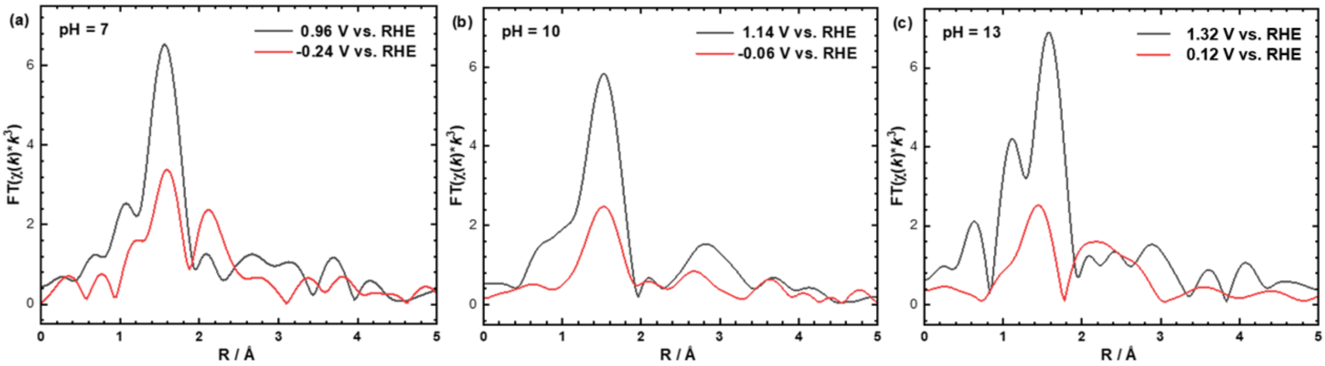


**Figure S14.** FT-EXAFS spectra of CuHdatrz/KB collected at (a) pH 7, (b) pH 10, and (c) pH 13.

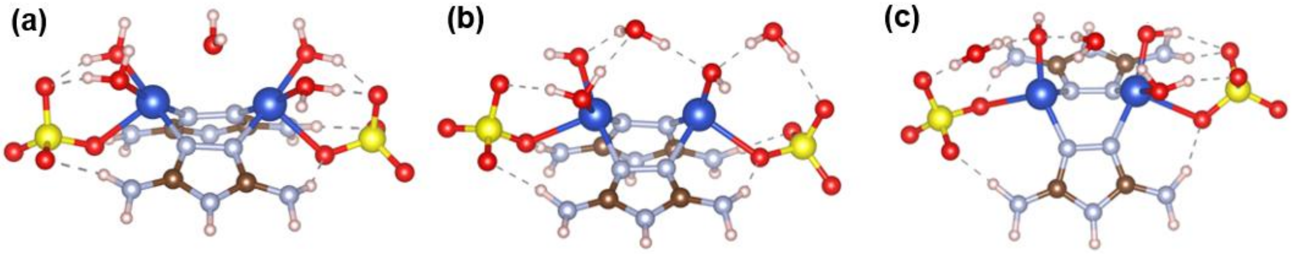


**Figure S15**. The optimized structures of CuHdatrz before and after the reduction: (a) the initial CuHdatrz with two $Cu^{II}$ ions and five $H_2O$ ligands (the total charge: 0) and (b,c) CuHdatrz with two $Cu^{I}$ ions and two OH and three $H_2O$ ligands (the total charge: –2). The Cu–Cu distances were calculated to be 3.485 Å for (a), 3.168 Å for (b) and 3.022 Å for (c).

**Table S1.** Comparison of e-$N_2$ORR activity and selectivity between previously reported (electro)catalysts and CuHdatrz used in this work.

| Catalyst | Type | Conditions | Temperature | Performances | Ref |
|---|---|---|---|---|---|
| Co-Clam | Electrocatalyst | -1.35 V vs. $Fc/Fc^+$, 1.0 μmol in 0.2 mL TBAP/benzonitrile, $N_2O$ sat'd | R.T | $N_2$ confirmed as sole product, FE and TOF not reported | 19 |
| Ni-[15]ane$N_4$ | Electrocatalyst | -0.95 V vs. NHE, 20 μM in 0.1 M KCl, Hg electrode, $N_2O$ sat'd | R.T | FE = 96 % TOF = 476 $h^{-1}$ | 20 |
| Co-TAPc | Electrocatalyst | -0.35 V ~ 1.10 V vs. SCE, 1.5 × $10^{-10}$ mol·$cm^{-2}$ drop-cast on basal plane graphite in BR buffer solution with 0.1M $NaClO_4$, $N_2O$ sat'd | R.T | $N_2$ confirmed as sole product, FE and TOF not reported | 21 |
| Ru-N($SiMe_2CH_2PtBu_2$) | Homogeneous catalyst | 6.0 μmol of Ru-N($SiMe_2CH_2PtBu_2$) in 5 mL of $(CH_2)_4O$ under 3 bar $N_2O$ + 4 bar $H_2$ | 65 °C | TON = 8.7 $h^{-1}$ | 22 |
| Rh-Pt-Olefin | Homogeneous catalyst | 7.0 μmol of Rh-Pt-Olefin in 2 mL of $(CH_2)_4O$ under 2 bar $N_2O$ + 2 bar $H_2$ | R.T | TON = 12.2 $h^{-1}$ | 23 |
| Re-bpy | Electrocatalyst | -1.72 V vs. SCE, 0.1 M Re-bpy in $CH_3CN/H_2O$ = 90/10 with 0.1 M n-$Bu_4NPF_6$, $N_2O$ sat'd | R.T | FE = 102 % TOF = 100 $h^{-1}$ | 24 |
| Ir-MesCNP | Homogeneous catalyst | 1.6 μmol of Ir-MesCNP in 0.6 mL of $(CH_2)_4O$ under 1 bar $N_2O$ + 1 bar $H_2$ | 55 °C | TON = 16.4 $h^{-1}$ | 25 |
| Cu-MeIm$_4$P$_2$Py | Electrocatalyst | −2.3 V vs. $Ag^+/Ag$, 1 mM in 0.1 M n-$Bu_4PF_6$ in MeCN with 100 mM $H_2O$, $N_2O$ sat'd | R.T | FE = 54 % TOF = 83 $h^{-1}$ | 26 |
| Fe-TPP | Electrocatalyst | −2.3 V vs. $Fe(C_5H_5)_2^{+/0}$, 1 mM in 0.1 M n-$Bu_4NPF_6$ in THF, $N_2O$ sat'd | R.T | FE = 100 ± 5 % TOF = 3.93 $h^{-1}$ | 27 |
| CuHdatrz | Electrocatalyst | −0.3 V vs. RHE, 2 μM Cu-Hdatrz drop-cast on 12.5 $cm^2$ carbon paper in 28.5 mL 0.04 M BR buffer solution with 0.1M $NaClO_4$, $N_2O$ sat'd | R.T | FE = 100 % TOF = 110 $h^{-1}$ | This work |
| CuHdatrz | Electrocatalyst | −0.6 V vs. RHE, 2 μM Cu-Hdatrz drop-cast on 12.5 $cm^2$ carbon paper in 28.5 mL 0.04 M BR buffer solution with 0.1M $NaClO_4$, $N_2O$ sat'd | R.T | FE = 67 % TOF = 656 $h^{-1}$ | This work |

**Table S2.** Fitted parameters of curve-fitting analysis of the FT-EXAFS oscillation of CuHdatrz/KB. The errors are shown in the brackets. The values without errors are fixed for curve-fitting analysis.

| *E* / V vs. RHE | | Cu–X | C.N.[a)] | *R*[b)] / Å | ss[c)] / Å$^2$ | R-factor |
|---|---|---|---|---|---|---|
| | 0.96 | N | 3.8 | 1.95 | 0.007(1) | 0.03 |
| pH 7 | -0.24 | N | 1.3 | 1.88±0.01 | 0.004(1) | 0.035 |
| | | Cu | 0.5(1) | 2.57±0.03 | 0.005 | |
| pH 10 | 1.14 | N | 3.8(2) | 1.93±0.01 | 0.0065 | 0.007 |
| | -0.06 | N | 1.9(2) | 1.86±0.02 | 0.0065 | 0.03 |
| | 1.32 | N | 4.0(3) | 1.91±0.01 | 0.0063 | 0.02 |
| pH 13 | 0.12 | N | 1.5 | 1.87±0.02 | 0.007(1) | 0.038 |
| | | Cu | 0.5 | 2.58±0.01 | 0.004(1) | |

a) Coordination numbers. b) Bond lengths. c) Debye-Waller factors.

## References


[1] M. Kato, K. Kimijima, M. Shibata, H. Notsu, K. Ogino, K. Inokuma, N. Ohta, H. Uehara, Y. Uemura, N. Oyaizu, T. Ohba, S. Takakusagi, K. Asakura, I. Yagi, *Phys Chem Chem Phys* **2015**, *17*, 8638-8641.

[2] U. O. Nwabara, A. D. Hernandez, D. A. Henckel, X. Chen, E. R. Cofell, M. P. de-Heer, S. Verma, A. A. Gewirth, P. J. A. Kenis, *ACS Appl Energy Mater* **2021**, *4*, 5175–5186.

[3] Y. Zhuang, Y. Iguchi, T. Li, M. Kato, Y. A. Hutapea, A. Hayashi, T. Watanabe, I. Yagi, *ACS Catal* **2024**, *14*(3), 1750-1758.

[4] T. Taguchi, T. Ozawa, H. Yashiro, *Phys Scr* **2005**, *T115*, 205-206.

[5] J. W. Cook, Jr., D. E. Sayers, *J Appl Phys* **1981**, *52*, 5024-5029.

[6] M. Newville, *J. Phys Conf Ser.* **2013**, *430*, 012007.

[7] A. L. Ankudinov, A. I. Nesvizhskii, J. J. Rehr, *Phys Rev B* **2003**, *67*, 115120.

[8] Larch: Data Analysis Tools for X-ray Spectroscopy, "XAFS: Fitting XAFS to Feff Paths," can be found under https://xraypy.github.io/xraylarch/xafs_feffit.html

[9] *Standard Methods for the Examination of Water and Wastewater*, 19th ed. and earlier editions; *American Public Health Association: Washington, DC, 1999.*

[10] M. J. Frisch, G. W. Trucks, H. B. Schlegel, G. E. Scuseria, M. A. Robb, J. R. Cheeseman, G. Scalmani, V. Barone, B. Mennucci, G. A. Petersson, H. Nakatsuji, M. Caricato, X. Li, H. P. Hratchian, A. F. Izmaylov, J. Bloino, G. Zheng, J. L. Sonnenberg, M. Hada, M. Ehara, K. Toyota, R. Fukuda, J. Hasegawa, M. Ishida, T. Nakajima, Y. Honda, O. Kitao, H. Nakai, T. Vreven, J. A. Montgomery Jr., J. E. Peralta, F. Ogliaro, M. Bearpark, J. J. Heyd, E. Brothers, K. N. Kudin, V. N. Staroverov, R. Kobayashi, J. Normand, K. Raghavachari, A. Rendell, J. C. Burant, S. S. Iyengar, J. Tomasi, M. Cossi, N. Rega, J. M. Millam, M. Klene, J. E. Knox, J. B. Cross, V. Bakken, C. Adamo, J. Jaramillo, R. Gomperts, R. E. Stratmann, O. Yazyev, A. J. Austin, R. Cammi, C. Pomelli, J. W. Ochterski, R. L. Martin, K. Morokuma, V. G. Zakrzewski, G. A. Voth, P. Salvador, J. J. Dannenberg, S. Dapprich, A. D. Daniels, Ö. Farkas, J. B. Foresman, J. V. Ortiz, J. Cioslowski, D. J. Fox, *Gaussian 09, Revision D.01, Gaussian, Inc., Wallingford CT, 2009.*

[11] Y. Zhao, D. G. Truhlar, *Theor Chem Acc.* **2008,** *120*, 215-241.

[12] M. Dolg, U. Wedig, H. Stoll, H. Preuss, *J Chem Phys,* **1987,** *86*, 866–872.

[13] E. Aznar, S. Ferrer, J. Borrás, F. Lloret, M. Liu-González, H. Rodríguez-Prieto, S. García-Granda, *Eur J Inorg Chem* **2006**, 5115-5125.

[14] S. Miertuš, E. Scrocco, J. Tomasi, *Chem Phys* **1981,** *55* (1), 117-129.

[15] M. Cossi, V. Barone, R. Cammi, J. Tomasi, *Chem Phys Lett* **1996,** *255* (4-6), 327-335.

[16] B. Mennucci, J. Tomasi, *J Chem Phys* **1997,** *106* (12), 5151-5158.

[17] B. Mennucci, E. Cancès, J. Tomasi, *J Phys Chem B* **1997,** *101* (49), 10506-10517.

[18] J. Tomasi, B. Mennucci, R. Cammi, *Chem Rev* **2005**, *105* (8), 2999-3093.

[19] J. P. Collman, M. Marrocco, C. M. Elliott, M. L'Her, *J Electroanal Chem* **1981**, *124*, 113-131.

[20] I. Taniguchi, T. Shimpuku, K. Yamashita, H. Ohtaki, *J Chem Soc Chem Commun* **1990**, *13*, 936-938.

[21] J. Zhang, Y.-H. Tse, A. B. P. Lever, W. J. Pietro, *J Porphyrins Phthalocyanines* **1997**, *1*, 323-331.

[22] R. Zeng, M. Feller, Y. Ben-David, D. Milstein, *J Am Chem Soc* **2017**, *139*, 5720-5723.

[23] P. Jurt, A. S. Abels, J. J. Gamboa-Carballo, I. Fernández, G. Le Corre, M. Aebli, M. G. Baker, F. Eiler, F. Müller, M. Wörle, R. Verel, S. Gauthier, M. Trincado, T. L. Gianetti, H. Grützmacher, *Angew Chem Int Ed* **2021**, *60*, 25372-25380.

[24] R. Deeba, F. Molton, S. Chardon-Noblat, C. Costentin, *ACS Catal* **2021**, *11*, 6099-6103.

[25] I. Ortega-Lepe, P. Sánchez, L. L. Santos, P. Lara, N. Rendón, J. López-Serrano, V. Salazar-Pereda, E. Álvarez, M. Paneque, A. Suárez, *Inorg Chem* **2022**, *61*, 18590-18600.

[26] J. L. Martinez, J. E. Schneider, S. W. Anferov, J. S. Anderson, *ACS Catal* **2023**, *13*, 12673-12680.

[27] J. S. Stanley, X. S. Wang, J. Y. Yang, *ACS Catal* **2023**, *13*, 12617-12622.
